# The Potential Role of Liver Transplantation as a Treatment Option in Colorectal Liver Metastases

**DOI:** 10.1155/2018/8547940

**Published:** 2018-01-17

**Authors:** Pål-Dag Line, Morten Hagness, Svein Dueland

**Affiliations:** ^1^Department of Transplantation Medicine, Oslo University Hospital, Oslo, Norway; ^2^Institute of Clinical Medicine, University of Oslo, Oslo, Norway; ^3^Department of Oncology, Oslo University Hospital, Oslo, Norway

## Abstract

Liver resection is the only potentially curative treatment option in patients with liver metastases from colorectal cancer, but only about 20% of the patients are resectable. Liver transplantation of patients with unresectable liver metastases was attempted in the early era but it was abandoned due to poor survival. During the last decade, several case reports, a controlled pilot study, and a retrospective cohort study indicated that prolonged disease-free survival and overall survival can be obtained in a proportion of these patients. Strict selection criteria have not yet been well defined, but tumor load, response to chemotherapy, pretransplant carcinoembryonic antigen level, and time interval from resection of the primary tumor to transplant are all factors related to outcome. Carefully selected patients may obtain 5-year overall survival that approaches conventional indications for liver transplant. The scarcity of liver grafts is a significant problem, but this can possibly to some extent be addressed by use of extended criteria grafts and novel surgical techniques. There is an increasing interest in liver transplantation in these patients in the transplant community, and currently 4 clinical trials are active and are recruiting.

## 1. Introduction

Colorectal cancer is the third most common cancer in men and the second most prevalent cancer in women and thus is a leading cause of cancer-related morbidity and death [1]. Over 50% of the patients develop distant metastasis, and the most frequent site is the liver. At the time of diagnosis, synchronous liver metastases are found in about 20–25% of the patients, and another 30% develop liver metastases at later stages [2].

Liver resection is the only potential curative therapeutic option for colorectal liver metastases (CRLM), and overall survival at 5 years has been reported to be between 20 and 58%, depending on clinicopathological features and patient selection [3, 4]. Although there have been substantial developments with respect to resectability and significant improvements regarding available chemotherapeutic possibilities, only around 20% of the patients with CRLM can be offered resection with curative intent.

Liver transplantation is an established therapeutic option in selected patients with primary liver tumors like HCC [5] and encouraging results have also been reported by applying rigorous protocols combining chemoradiation and transplantation in highly selected patients with hilar cholangiocarcinoma [6]. For secondary liver tumors, metastasis from neuroendocrine tumors with low proliferation rate (WHO grade 1, KI-67 less than 3–5%) is an emerging indication [7, 8]. Concerning CRLM, liver transplantation could be regarded as the “ultimate” liver resection. On this background, there have been attempts during the evolvement of liver transplantation to utilize transplantation as treatment of unresectable CRLM.

The aim of the present review is to present an update of current knowledge within the field, emphasizing recent reports and developments.

## 2. Historical Overview

Early attempts to transplant patients with CRLM were performed in the very infancy of liver transplantation [9, 10]. The operations were done at a time when prolonged postoperative survival was a rarity due to severe complications and lack of effective immunosuppression. The first, and to date, largest published series was performed at the university of Vienna. 25 patients were transplanted and the reported overall survival [OS] at 1, 3, and 5 years was 76%, 32%, and 12%, respectively [11, 12]. Due to poor OS, offering liver transplantation to these patients was abandoned by the Vienna group, and colorectal liver metastasis was internationally considered a contraindication. Hoti and Adam have provided a brief account for the experiences from the European Liver Transplant Registry (ELTR), where a total of 55 patients were registered until 2007. The 25 patients transplanted in Vienna are included in this cohort. One-year OS and five-year OS were 62% and 18%, respectively [13]. 80% of the procedures were performed before 1995. It is worth noting that in this particular time period selection criteria for transplant were less developed, immunosuppression protocols were variable, and many of the centres had low volume experience. The postoperative mortality after liver transplantation in general was far higher than is the case in the current era. The cause of death in 44% of the patients was not related to cancer recurrence. On this background, the early results of liver transplantation for CRLM cannot be fully evaluated in terms of efficacy.

Long-term survival was observed in 2 patients from the ELTR database (9 and 21 years) [13], and later other reports on treatment success in a single patient or a few patients have been published [14–16].

## 3. Recent Published Experiences

The first controlled trial on liver transplantation for CRLM was started in Oslo in 2006 (SECA Study). Norway is a low-incidence country for HCV infection, HCC, and alcoholic hepatitis and has benefitted from a donation rate usually above 20 pr. million inhabitants. Thus, the waiting times have been short and the mortality on the waiting list was below 3%. This provided an opportunity to explore liver transplantation of CRLM scientifically, without the risk of negatively impacting other patients on the waiting list. 21 patients were transplanted, and the estimated 5-year OS was 60% after median 27 months of follow-up and with a disease-free survival of 35% at one year [17]. It is important to view the results from the SECA trial as a “proof of concept” study. The initial inclusion criteria were quite strict, with the intention of being able to select optimal candidates from an oncological point of view for the procedure. Due to the novel and provocative nature of the study concept, it was hard to recruit patients at the outset of the study. Therefore, it was decided to amend and simplify the inclusion criteria so that a broader proportion of patients could be considered for inclusion. The final study population was therefore quite heterogeneous with respect to chemotherapy lines given, chemotherapy response, tumor load, and parameters related to tumor biology. The included patients consisted of 11 colon cancers and 10 rectum primaries: 16 patients had T3 tumors, 3 were T4, and the remaining 2 had T2. Median number of CRLM was 8 (range: 4–40), with a median diameter of 4,5 cm (range: 2,8–13,9). All patients had received chemotherapy (first, second, and third line in 9, 8, and 4 patients, resp.), and pretransplant carcinoembryonic antigen (CEA) ranged from 1 to 2001 *μ*g/l (median: 15) [17]. If the patients had been resectable given these characteristics, Fong risk scoring would have yielded high risk in 16 and low risk in 5 patients, which typically translates to 5-year disease-specific survival following liver resection with curative intent of 20−25% [18, 19]. Thus, the overall survival from an oncological perspective was impressive. Disease-free survival was, however, short and the recurrence rate “universal,” with a median time to relapse of 10 months [2–24]. Recurrence was defined as time from transplant to detection of metastasis or recurrence of the primary. The majority of the patients, 68%, relapsed in the lungs [20]. From the data, it is evident that an unknown proportion of the cases were a result of missed staging, where retrospective examination of undefinable small nodules on pretransplant CT scans of the thorax in 7/17 patients later proved to be lung metastases that were present before transplant [20]. The true “de novo” recurrence rate is therefore probably lower.

In a recent, uncontrolled, retrospective cohort study, the group from “Compagnons Hépato-Biliaires” have summarized their experiences in 12 patients [21]. Ten of the patients had undergone previous resection and 11/12 had received chemotherapy with response. Time from resection of the primary to transplantation was median 41 months [12–97] and preoperative CEA level was 2–314 *μ*g/l [median: 16,9]. Median number of lesions was 9, and only two patients had maximal diameter over 5 cm [5,5 and 8 cm]. Overall patient survival was 83%, 62%, and 50% at 1, 3, and 5 years, respectively. Six patients were diagnosed with recurrence, 5 with lung metastases, 3 with liver metastases, and 1 with peritoneal metastasis. Disease-free survival at 1, 3, and 5 years was 56%, 38%, and 38%, respectively. Although this retrospectively collected cohort is not a controlled study and the patients are highly selected through a long interval from resection of the primary to transplant, the outcomes with respect to overall survival are similar to the SECA 1 trial. Furthermore, the paper demonstrates, like previous case reports have done [14–16], that disease-free survival may be obtained.

## 4. Recurrence and Disease-Free Survival

Recurrence after liver transplantation for CRLM seems to present in two forms, with clear distinction with respect to long-term prognosis. Metastases to the transplanted liver or other nonpulmonary recurrences are associated with decreased overall survival and most likely are sign of systemic disease [20]. From the SECA study and the report from Toso et al. [21], it is evident that the majority of the relapses in both studies were lung metastases. It is worth noting that in the SECA trial the pulmonary metastases were slow-growing, even though no specific chemotherapy or any other treatment was given. A large proportion of the patients could be offered lung resection and obtain status of no evidence of disease [20]. From the transplant literature, it is well documented that transplant recipients have an increased risk of de novo cancer, and this has been attributed to chronic immunosuppression [22–24]. Thus, the highly relevant concern that relapses in these patients could display an accelerated and more aggressive growth pattern has been raised. The clinical experience from the trials conducted in Oslo does not fully confirm this. In a recent study, the growth rate of pulmonary metastases in patients transplanted for CRLM has been compared with a group of patients with rectal cancer and pulmonary metastases only [25]. The results indicated that the pulmonary lesions in the transplant group displayed a growth rate (given as tumor volume doubling time) that was equal to that of the nonimmunosuppressed patients (median 124 versus median 110 days). This is contrary to what one might expect and an important result when interpreting the results after liver transplantation for CRLM.

Another finding elucidating the impact of immunosuppression and progressive disease is the comparative study between chemotherapy and transplantation utilizing data from the NORDIC VII Trial [26]. The patients treated with first-line chemotherapy and the transplanted cohort had similar DFS (8 and 10 months, resp.), but the study showed a dramatic difference in overall survival after recurrence. The SECA patients had a 5-year OS of 53% after detection of recurrence, whereas this was 6% in the NORDIC VII patients. Even patients with progression on second-line and third-line chemotherapy at the time of transplant had a far better outcome than standard of care chemotherapy, suggesting a fundamental impact on survival of removing liver metastases in these patients [27].

## 5. Patient Selection and Outcome

Although previous studies indicate that most CRLM patients will experience a substantial survival benefit from liver transplantation, improved patient selection is crucial if transplantation of this patient group is going to become a clinical reality. From the SECA 1 trial, four clinical factors emerged as predictive of poor survival:

- Diameter of the largest tumor ≥ 55 mm
- Pretransplant CEA level > 80 *μ*g/l
- Progressive disease on chemotherapy
- Time from resection of the primary tumor to transplant < 2 years

These are all factors well known for risk scoring in liver resection [18, 28], and patients having all these factors at time of liver transplantation were shown to be a high risk group with short overall survival [17]. In the recent study by Toso et al., both time from surgery of the primary to transplant > 24 months and CEA level < 80 *μ*g/l were related to disease-free survival [21]. They were not able to show a similar relationship between DFS and maximal tumor diameter or disease progression. This is not unexpected, since patients with signs of progressive disease were not included, and the limitations in statistical power due to small sample size (*n* = 12) might have failed to reveal an association between tumor size and DFS.

The survival benefit of transplantation and impact of improved selection have been highlighted in a recent study, where DFS and OS after liver transplantation of high-risk and low-risk CRLM patients (according to the four criteria discussed above) were compared with liver transplantation of HCC patients within and outside the Milan criteria. The CRLM patients had a higher tumor load (median 8 lesions) than the HCC patients (median 1 lesion). DFS was shorter in both high-risk and low-risk CRLM groups compared with HCC patients. The 5-year OS in low-risk CRLM patients was 75% compared to 76% and 56% in HCC patients within and beyond the Milan criteria. The 4 patients with metachronous CRLM were all alive at 5 years after transplant. When survival after recurrence was compared between CRLM and HCC, 89% of patients with recurrent HCC were dead within 20 months, whereas 86% of the low-risk CRLM patients were alive at 24 months. This clearly underlines the impact of patient selection and might also suggest that patients with CRLM might have a favourable outcome compared with patients transplanted for HCC outside the Milan criteria.

A new study on liver transplantation for unresectable colorectal liver metastases was initiated in Oslo in 2011 (SECA 2 study, clinicaltrials.gov: NCT01479608). In this protocol, more stringent selection criteria have been utilized, and the analysis of results is underway and will be published shortly. Preliminary findings indicate that further improvements in disease-free survival and overall survival may be anticipated.

Improvements in preoperative imaging studies could be a crucial factor to select the best candidates for liver transplantation for CRLM. All patients in Oslo have been investigated by computed tomography (CT) scans of the chest and abdomen/pelvis as well as F-18 fluorodeoxyglucose positron emission tomography (^18^F-FDG PET) in combination with CT prior to liver transplantation. PET techniques have been used extensively in diagnosis and staging of malignant tumors [29]. In a recent study, we hypothesized that volumetric and metabolic PET parameters before transplant could be related to posttransplant survival outcome. The results from this study indicate that metabolic tumor volume (MTV) and total lesion glycolysis (TLG) prior to transplant were both predictive of improved overall survival [30]. Thus, this diagnostic modality should be an integral part of the selection algorithm before transplant.

## 6. Limitations Related to Liver Graft Availability

A fundamental limitation to continued studies on liver transplantation for CRLM at an international level as well as introducing the concept into the clinic on a limited scale is the scarcity of liver grafts, which is a harsh reality in most countries. Allocating livers to patients with colorectal liver metastases cannot negatively impact the access to liver transplantation for patients with conventional transplant indications. Since suitable patients with unresectable liver metastases do not have liver insufficiency or portal hypertension, the requirements with regard to organ quality might be lower and the tolerability for extended criteria crafts [31] could be better than in patients with chronic liver failure. Hence the use of extended criteria grafts that are not routinely used could be one way to expand the donor pool. In Oslo, a protocol for randomization between liver transplantation and best oncologic treatment utilizing this type of grafts is approved and initiated. Another approach is the so-called RAPID concept (Resection and Partial Liver Segment 2/3 Transplantation with Delayed Total Hepatectomy). The concept entails a two-step procedure, where step 1 consists of liver resection of the left liver combined with transplantation of a segment 2+3 graft and modulation of graft portal inflow [32]. This induces fast regeneration of the transplanted graft during the course of 2-3 weeks and is followed by delayed total hepatectomy of the liver remnant as stage 2. The RAPID concept is currently evaluated in a prospective pilot study in Oslo (clinicaltrials.gov: NCT02215889). Although the preliminary results are promising, they are too early to conclude with regard to the future role of this approach in clinical practice. This has, understandably, due to the controversial nature, initiated some controversy [33]. One potential possibility that could have huge implications on clinical practice if the RAPID concept can be developed successfully is to use segment 2+3 from living donors. Some cases have already been performed in Germany (A. Königsrainer and U. Settmacher, personal communication). This could greatly improve the access to liver grafts for a proportion of potentially eligible patients with unresectable liver only metastases, but further studies are needed before this can become a clinical reality.

## 7. Conclusion

The current status of knowledge suggests that it is possible to obtain prolonged disease-free survival and overall survival after liver transplantation for unresectable colorectal liver metastases. Stringent patient selection is mandatory and crucial in order to obtain results that could justify allocation of liver grafts to these patients. Although optimal selection criteria are still not sufficiently clarified, low-risk CRLM patients can most likely obtain overall survival that is comparable to that observed in patients transplanted for HCC. The benefit of liver transplantation for CRLM patients is very high, given that palliative chemotherapy is the only alternative. The access to liver grafts for this extended indication can be improved by utilizing extended criteria donor grafts and possibly novel surgical approaches such as the RAPID concept. At present, four clinical trials on liver transplantation for unresectable colorectal liver metastases are registered at https://clinicaltrials.gov [12]. It is important that all transplantations on this indication are performed within the setting of prospectively controlled studies. Furthermore, a separate international registry collecting the international experience within this field would be of high scientific value and could further facilitate international collaboration.
